# Reproducibility and Relative Validity of Food Group Intake in a Food Frequency Questionnaire Developed for the Tehran Lipid and Glucose Study

**DOI:** 10.2188/jea.JE20090083

**Published:** 2010-03-05

**Authors:** Firoozeh Hosseini Esfahani, Golaleh Asghari, Parvin Mirmiran, Fereidoun Azizi

**Affiliations:** 1 Obesity Research Center, Research Institute for Endocrine Sciences, Shahid Beheshti University of Medical Sciences, Tehran, I.R. Iran; 2 Department of Human Nutrition, Faculty of Nutrition and Food Technology, National Nutrition and Food Technology Institute, Shahid Beheshti University of Medical Sciences, Tehran, I.R. Iran; 3 Endocrine Research Center, Research Institute for Endocrine Sciences, Shahid Beheshti University of Medical Sciences, Tehran, I.R. Iran

**Keywords:** food frequency questionnaire, validity and reproducibility, food group, Tehran

## Abstract

**Objective:**

To examine the validity and reproducibility of food groups in the semi-quantitative food frequency questionnaire (FFQ) developed for the Tehran Lipid and Glucose Study (TLGS).

**Methods:**

To evaluate the reproducibility of food groups included in the FFQ, 132 subjects (61 men and 71 women) aged 20 years or older twice completed a 168-item FFQ (FFQ1, FFQ2), with a 14-month interval between FFQ1 and FFQ2. Over the 1-year interval, 12 dietary recalls (DRs) were collected (1 each month) to assess the validity of the FFQ. Seventeen food groups were derived from the FFQ based on methods described in previous studies. Age-adjusted and deattenuated Spearman correlation coefficients were used to assess the validity of the FFQ.

**Results:**

The mean (SD) age and body mass index of subjects were 35.5 (16.8) years and 25.5 (5.2) kg/m^2^, respectively. Validity correlation coefficients ranged from 0.03 (liquid oil) to 0.77 (simple sugars) in men (median, 0.44), and from 0.12 (snacks) to 0.79 (simple sugars) in women (median, 0.37). The energy-adjusted intraclass correlation coefficient, which reflects the reproducibility of the FFQ, was 0.51 in men and was highest for tea and coffee (0.91); in women it was 0.59 and was highest for simple sugars (0.74). The highest percentage of complete agreement and disagreement was observed for snacks and desserts (60.6%) and potatoes and dairy products (12.8%), respectively, in men, and tea and coffee (62.9%) and legumes (15.7%) in women.

**Conclusions:**

The FFQ that was designed for the TLGS was found to be reliable and valid for assessing the intake of several food groups.

## INTRODUCTION

As the third millennium of the Common Era commences, the high prevalence of obesity is fast becoming a major public health concern in most countries. This phenomenon has been associated with an epidemic outbreak of chronic diseases such as diabetes, cardiovascular disease, and cancers,^1^^,^^2^ diseases that are designated as preventable because they can be avoided or managed by certain changes in individuals’ lifestyle patterns.^3^ One such change is dietary factors. Several prospective studies have examined the association between various food groups (eg, grains, fruits, vegetables, milk, and meat products) and the incidence of chronic diseases.^4^^–^^6^ As a general epidemiological principle, long-term exposure to a diet is more important than dietary intake on a few specific days.^7^

Because food frequency questionnaires (FFQs) are easy and cheap to administer, they have proven to be an extremely practical tool for dietary assessment in epidemiological studies.^7^ Since FFQs are used for ranking individuals based on their habitual intakes of foods and nutrients, accurate estimation of intakes is crucial.^8^ As in other dietary assessment methods, random and systematic errors can arise in FFQ estimates, which may not represent the “actual” usual diet.^9^ Random errors that occur in FFQs can attenuate the associations in epidemiological studies.^10^^,^^11^ To prevent incorrect estimations of dietary intakes, which may lead to misunderstandings of the relationship between dietary factors and diseases, the reproducibility and validity of an FFQ is assessed.^12^ Hence, surveys using an FFQ as their dietary assessment tool must validate their measurement method for nutrients, foods, and food groups.^13^^–^^20^

Population-based surveys in Tehran, the capital city of Iran, have revealed an increase in the rate of chronic diseases.^21^ The prevalences of obesity (29.5% in women vs 14.4% in men) and metabolic syndrome (30% in adults) were reported to be high.^22^^,^^23^ The Tehran Lipid and Glucose Study (TLGS) is an ongoing population-based prospective cohort study investigating non-communicable chronic disease risk factors in Iran, for which a 168-item FFQ was designed.^24^ In the present study, we examined the validity and reproducibility of this FFQ for 17 food groups.

## METHODS

### Study population

The subjects of the present study are a subsample drawn from the TLGS, an urban population based in district 13 of Tehran, capital of the Islamic Republic of Iran.^24^

An age- and sex-stratified sample consisting of 200 subjects, aged 20 to 70 years, was selected from the 15 005 participants of the TLGS (1999–2001). Subjects were proportionately distributed across five 10-year age groups and 2 sexes to generalize the results to all age groups and both sexes. The inclusion criteria were current residence in Tehran for longer than 3 years and no history of diabetes or renal or liver disease. Of a total of 200 potential participants, 162 agreed to participate in this study (response rate: 81%). We excluded participants who did not satisfactorily complete the FFQs (*n* = 12), had more than 2 missing 24-hour dietary recalls (DRs) (*n* = 15), or who received a diagnosis of a chronic disease during the study period (*n* = 3), after which the final population was 132 subjects (61 males and 71 females). BMI, education level, smoking status, total cholesterol, and systolic and diastolic blood pressure were then recorded. This study was approved by the ethics committee of the Research Institute for Endocrine Sciences of the Shahid Beheshti University of Medical Sciences; informed written consent was obtained from each subject.

### Study design

Data collection began in 2002 and continued for the subsequent 14 months. During the study period, twelve 24-hour DRs were collected from each participant. An FFQ was administered 1 month before collection of the first 24-hour DR (FFQ1), and a second was administered 1 month after the last 24-hour DR (FFQ2). The study design is shown in the Figure.

**Figure 1.**
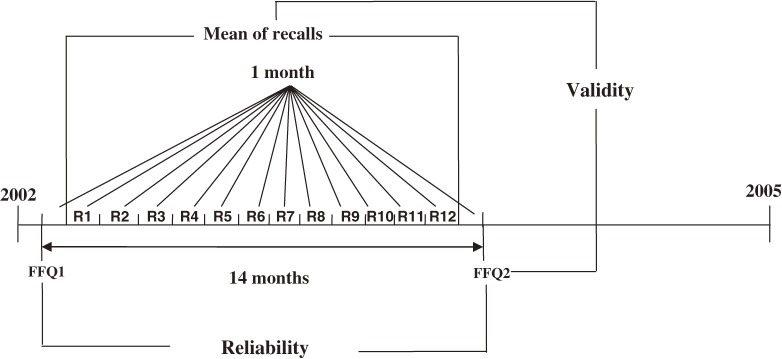
The study design used to test the relative validity and reliability of the Food Frequency Questionnaire developed for the Tehran Lipid and Glucose Study (TLGS). R = 24-hour dietary recall, FFQ = Food Frequency Questionnaire. FFQ1 and FFQ2 were completed 1 month before the first recall and 1 month after the 12th recall, respectively; twelve 24-hour dietary recalls were collected on a consecutive monthly basis. The second follow-up survey of the TLGS began in 2003 and was completed in 2005.

### Food frequency questionnaire and 24-hour dietary recall

The FFQ, originally developed for the TLGS, was a Willett-format questionnaire modified based on Iranian food items^25^ and contains questions about average consumption and frequency for 168 food items during the past year.^7^ The food items were chosen according to the most frequently consumed items in the national food consumption survey in Iran.^25^ Because different recipes are used for food preparation, the FFQ was based on food items rather than dishes, eg, beans, different meats and oils, and rice. Subjects indicated their food consumption frequencies on a daily basis (eg, for bread), weekly basis (eg, for rice and meat), monthly basis (eg, for fish), yearly basis (eg, for organ meats), or a never/seldom basis according to portion sizes that were provided in the FFQ. For each food item on the FFQ, a portion size was specified using USDA serving sizes (eg, bread, 1 slice; apple, 1 medium; dairy, 1 cup) whenever possible; if this was not possible, household measures (eg, beans, 1 tablespoon; chicken meat, 1 leg, breast, or wing; rice, 1 large, medium, or small plate) were chosen. Table 1 shows food items and portion sizes used in the FFQ. Trained dietary interviewers with at least 3 of experience in the Nationwide Food Consumption Survey project^25^ or TLGS^26^ administered the FFQs and 24-hour DRs during face-to-face interviews. The interviewer read out the food items on the FFQ, and recorded their serving size and frequency. The interview session took about 45 minutes. The interviewer for FFQ1 and FFQ2 was the same for each participant. Daily intakes of each food item were determined based on the consumption frequency multiplied by the portion size or household measure for each food item.^27^ The weight of seasonal foods, like some fruits, was estimated according to the number of seasons when each food was available.

**Table 1.** Food grouping used in the study of validity and reliability of the food frequency questionnaire developed for the TLGS^a^

Food groups	Food items (*n* = 168)	Portion size
1. Whole grains	All whole and dark breads (Barbari, Sangak, Taftoon, and ​ Toasted bread (whole grain))	Slice
	Popcorn	Cup
	Cooked barley, bulgur	Tablespoon
	Corn	One medium
	Biscuits prepared with whole grains	Number
2. Refined grains	White bread (Lavash)	Slice
	Baguette	Number
	Cooked rice and pasta	Plate
	Cooked angel hair pasta, reshteh, and wheat flour	Cup
3. Potatoes	French fries, baked potatoes	Number
4. Dairy products	All kinds of milks (whole, low fat, skim, cocoa and chocolate), ​ doogh (yogurt drink)	Cup
	Yogurt (plain and whole)	
	Yogurt (Concentrated and creamy), cream, kashk	Tablespoon
	Cheese (plain and creamy)	
	Ice cream (plain and traditional (high fat))	Half of a cup
5. Vegetables	Raw and cooked leafy vegetables, shredded lettuce, celery, ​ green pea, spinach, mushroom	Cup
	Raw and cooked tomato, cucumber, squash, eggplant, carrot, ​ garlic, onion, green pepper, turnip, green chilies	Number
	Cooked green bean, fried onion	Tablespoon
	Cruciferous vegetables (cauliflower, red and white cabbage)	
	Pumpkin	Slice
6. Fruits	Cantaloupe, Persian melon, watermelon	Slice
	Pear, apricot, apple, cherry, peach, nectarine, green plum, fig, grapes, ​ kiwi, grapefruit, orange, persimmon, tangerine, pomegranate, dates, ​ prune (yellow and red), sour cherry, strawberry, banana, sweet lemon, ​ lime lemon, mulberry, dried fruits (fig, mulberry, peach and apricot)	Number
	Cranberry, pineapple (raw and canned)	Cup
	Lime juice	Teaspoon
	Raisins	Tablespoon
	Canned fruits	Can
7. Legumes	Cooked lentil, bean, chickpea, cooked broad bean, soy bean, ​ Mung bean, split peas	Tablespoon
8. Meats	Tuna	Half a can
	Egg (all preparations), hamburger, sausage, organ meat ​ (brain, tongue, feet, and head)	Number
	Poultry, organ meat (liver, kidney and heart), tripe	Piece
	Bologna (beef), fish (all fishes except tuna)	Slice
	Ground meat	Tablespoon
	Red meats (beef, lamb)	Stew meat slice (ounce)
9. Nuts and seeds	Peanut, almond, walnut, pistachio, hazelnut	Number
	Seeds	
10. Solid fats	Hydrogenated fats, animal fats	Tablespoon
	Tallow (fat)	Piece
	Butter, hydrogenated margarine	Teaspoon
11. Liquid oils	All vegetable oils and olive oil, mayonnaise	Tablespoon
	Olives	Number
12. Tea and coffee	Tea and coffee	Cup
13. Salty snacks	Pickles in vinegar and salted vegetables	Tablespoon
	Salted pickles	Number
14. Simple sugars	Sugar	Teaspoon
	Cube sugar, noghl, and candy	Number
15. Honey and jams	Honey	Teaspoon
	Jams	Tablespoon
16. Soft drinks	All soft and sweet drinks, beer (non-alcoholic), syrup; ​ fruit juices (grapefruit, orange, apple and cantaloupe)	Cup
17. Snacks and desserts	Sponge cake, other cakes	Slice
	Yazdi cake (plain cake with rasins), chocolates, pastries (non-crème ​ and creamy), all biscuits other than those made from whole grain, ​ crackers, patties, gaz, sohan,	Number
	Crème caramel, halvah (homemade)	Tablespoon
	Puffs, potato chips and halvah (non-homemade)	Pocket

^a^TLGS – Tehran Lipid and Glucose Study.

The right column shows the attributed portion sizes for all 168 food items used in the food frequency questionnaire.

Dietary data were also collected monthly by means of twelve 24-hour DRs that lasted for 20 minutes on average. For all subjects, 2 formal weekend day (Thursday and Friday in Iran) and 10 weekdays were recalled. All recall interviews were performed at subjects’ homes to better estimate the commonly used household measures and to limit the number of missing subjects. Detailed information about food preparation methods and recipe ingredients were considered by interviewers. To prevent subjects from intentionally altering their regular diets, participants were informed of the recall meetings with dietitians during the evening before the interview. All recalls were checked by investigators, and ambiguities were resolved with the subjects. Mixed dishes in 24-hour DRs were converted into their ingredients according to the subjects’ report on the amount of the food item consumed, thus taking into account variations in meal preparation recipes. For instance, broth or soup ingredients—usually vegetables (carrot or green beans), noodles, barley, etc.—differed according to subjects’ meal preparation. Because the only available Iranian food composition table (FCT)^28^ analyzes a very limited number of raw food items and nutrients, we used the USDA FCT^29^ as the main FCT; the Iranian FCT was used as an alternative for traditional Iranian food items, like kashk, which are not included in the USDA FCT.

The food items on the FFQ and DR were grouped according to their nutrient contents, based on other studies,^30^ and modified according to our dietary patterns. Seventeen food groups were thus obtained, as follows: 1) whole grains, 2) refined grains, 3) potatoes, 4) dairy products, 5) vegetables, 6) fruits, 7) legumes, 8) meats, 9) nuts and seeds, 10) solid fat, 11) liquid oil, 12) tea and coffee, 13) salty snacks, 14) simple sugars, 15) honey and jams, 16) soft drinks, and 17) desserts and snacks (Table 1). The 168 food items on the FFQ were allocated to these 17 food groups, and the amounts in grams of each item were summed to obtain the daily intake of each food group.

### Statistical analysis

Medians were calculated for intakes of all food groups from both FFQs and 24-hour DRs. The Kolmogorov–Smirnov test and histograms were used to test the normality of mean food group intakes and variable distributions; the Wilcoxon signed rank test was used to determine any over- or underestimation between the mean intakes of 24-hour DRs and the FFQs. The Spearman correlation coefficient was used to analyze the variables for food group intakes from the FFQs and 24-hour DRs. The FFQ2 represents the time period during which DRs were collected. Using the residual method,^7^ energy- and age-adjusted food group intakes were calculated to exclude the possibility of variation due to differences in age and energy intake.^7^ Deattenuated correlation coefficients were reported using the Rosner and Willett formula to correct the within-person variations in 12-day recalls.^31^ Crude and energy-adjusted intraclass correlation coefficients were calculated to assess the reproducibility of the FFQ. The Statistical Package for Social Science (SPSS Inc, Chicago TL. Version 13) was used for all statistical analyses.

## RESULTS

A comparison of our study participants and the TLGS population is presented in Table 2. Sex, education level, and smoking status, as well as mean (SD) BMI, cholesterol, systolic and diastolic blood pressure, were similar between the groups. The median intake of 17 food groups measured by twelve 24-hour DRs and 2 FFQs, and the mean difference of each FFQ from 24-hour DRs are shown in Table 3 for both sexes. The mean values of the FFQ2 were significantly higher than the 24-hour DRs for dairy products, nuts and seeds, liquid oil, and salty snacks in men, and for refined grains, dairy products, nuts and seeds, tea and coffee, and salty snacks in women (*P* < 0.05). In contrast, underestimation was observed for meats and soft drinks in men and for soft drinks in women (*P* < 0.05). In comparison with the FFQ1, the FFQ2 tended to overestimate the consumption of legumes, nuts and seeds, liquid oil, and snack and dessert consumption in men, and dairy products in women; underestimation for refined grains and soft drinks was seen only in men (*P* < 0.05). In both sexes, the highest percentage of mean difference was seen for nuts in FFQ1 and for fruits in FFQ2.

**Table 2.** Characteristics of study participants and the TLGS^a^ population

Characteristics	Present study (*n* = 132)	TLGS (*n* = 15 005)
Age (yr)	43.3 (16.9)^b^	37.1 (13.8)
BMI^a^ (kg/m^2^)	25.1 ± 5.4	26.7 ± 5.0
Cholesterol (mg/dL)	202 ± 53	209 ± 47
Systolic BP^a^ (mm Hg)	113.4 ± 13.5	115.2 ± 18.9
Diastolic BP^a^ (mm Hg)	75.6 ± 9.9	75.7 ± 11.0
Sex (%)		
Male	46	42
Female	54	58
Education level (%)		
Non-academic	87.5	86.4
Academic	11.5	13.6
Smoking status (%)		
Smoker	10.7	13.7
Non-smoker	89.4	86.3

^a^TLGS – Tehran Lipid and Glucose Study; BMI – Body Mass Index; BP – Blood Pressure.

^b^Values are mean (standard deviation).

**Table 3.** Daily median intakes of 17 food groups and energy intake in both sexes, estimated by twelve 24-hour dietary recalls (DRs) and 2 food frequency questionnaires (FFQs) developed for the TLGS^a^

Food group (g)	Men (*n* = 61)					Women (*n* = 71)				
	
	24-h DRs	FFQ1		FFQ2		24-h DRs	FFQ1		FFQ2	
					
	Median	Median	%^b^	Median	%^b^	Median	Median	%^b^	Median	%^b^
Energy (kcal)	2359^c^	2733	14	2648^c^	12	1705	2040	25	2152^c^	26
Whole grains	84.2	83.9	7	105	16	65.1	59.5	17	67.5	18
Refined grains	459	506	13	443^d^	−6	297	281	11	330^c^	17
Potatoes	29.2	25.0	−3	28.0	0	21.9	27.8	24	20.9	6
Dairy products	273	361	39	318^c^	24	202	235	35	283^c,d^	60
Vegetables	213	208	12	217	8	225	212	8	223	10
Fruits	228	407	96	354	51	213	399	97	278	64
Legumes	28.6	22.2	−23	26.4^d^	0	28.4	15.9	−27	18.4	−13
Meats	115	92	−15	103^c^	−13	71.2	65.4	−3	64.6	0
Nuts and seeds	3.2	4.1	35	8.0^c,d^	123	3.1	3.2	74	3.5^c^	119
Solid fats	29.2	25.4	5	25.4	−10	22.1	23.5	11	22.8	11
Liquid oil	6.4	6.3	33	10.6^c,d^	84	6.6	4.9	0	6.9^d^	41
Tea and coffee	738	825	12	750	11	553	525	4	565^c^	11
Salty snacks	9.5	10.0	2	11.7^c^	25	10.7	9.6	35	11.9^c^	50
Simple sugars	39.3	35.2	−1	41.0	−4	25.2	19.7	−11	19.8	4
Honey and jams	4.1	3.4	8	4.9	68	3.9	1.5	13	1.5	13
Soft drinks	131.2	86.9	−25	69.9^c,d^	−42	58.5	56.5	−3	34.6^c^	−20
Snacks and desserts	13.9	24.4	47	28.3^d^	58	15.3	15.3	7	14.0	48

^a^TLGS – Tehran Lipid and Glucose Study; FFQ1 and FFQ2 were completed 1 month before the first recall and 1 month after the 12th recall, respectively; twelve 24-hour dietary recalls were collected on a monthly basis during the 12 months.

^b^percent of mean difference from 24-h DRs: [(FFQ − 24-h DRs)/24-h DRs] × 100.

^c^Different from 24-hour recalls comparing mean values, Wilcoxon signed rank test *P* < 0.05.

^d^Different from FFQ1 comparing mean values, Wilcoxon signed rank test *P* < 0.05.

The crude, energy-adjusted and deattenuated Spearman correlation coefficient was calculated to assess the validity of FFQs on food groups (Table 4). All crude correlations were greater than 0.3, except for potatoes, legumes, liquid oil, and salty snacks in men, and refined grains, potatoes, legumes, and nuts and seeds in women. The lowest and highest correlations between the FFQ2 and 24-hour DRs were seen for legumes and for tea and coffee, respectively, in both sexes. In men, adjusted and deattenuated Spearman correlation coefficients ranged from 0.09 (liquid oil) to 0.90 (tea and coffee) for FFQ1, and 0.03 (liquid oil) to 0.77 (simple sugars) for FFQ2. These values were the same in women and ranged from 0.12 (snacks and desserts) to 0.79 (simple sugars). All adjusted and deattenuated correlations were greater than 0.3 for FFQ1, except for liquid oil and salty snacks in men, and refined grains, potatoes, legumes, and nuts, and seeds in women. For FFQ2, the exceptions were legumes, solid fat, liquid oil, and salty snacks in men, and potatoes, legumes, salty snacks, and snacks and desserts in women.

**Table 4.** Correlation coefficients of mean food group intakes estimated using twelve 24-hour dietary recalls and FFQs developed for the TLGS in both sexes^a^

Food group	Men (*n* = 61)				Women (*n* = 71)			
	Spearman correlation coefficient^b^				Spearman correlation coefficient^c^			
	
	Crude		Adjusted and deattenuated^d^		Crude		Adjusted and deattenuated^d^	
			
	FFQ1	FFQ2	FFQ1	FFQ2	FFQ1	FFQ2	FFQ1	FFQ2
Whole grains	0.45	0.49	0.45	0.44	0.49	0.45	0.46	0.51
Refined grains	0.63	0.72	0.35	0.53	0.28	0.54	0.27	0.33
Potatoes	0.35	0.24	0.36	0.37	0.29	0.43	0.28	0.26
Dairy products	0.89	0.73	0.53	0.61	0.61	0.56	0.61	0.59
Vegetables	0.52	0.66	0.42	0.69	0.56	0.50	0.59	0.50
Fruits	0.59	0.71	0.48	0.71	0.37	0.31	0.49	0.35
Legumes	0.38	0.25	0.43	0.26	0.09	0.28	0.10	0.18
Meats	0.46	0.48	0.37	0.39	0.45	0.52	0.36	0.37
Nuts and seeds	0.53	0.51	0.58	0.54	0.28	0.38	0.27	0.39
Solid fats	0.57	0.48	0.32	0.10	0.34	0.49	0.45	0.33
Liquid oil	0.14	0.16	0.09	0.03	0.53	0.38	0.51	0.35
Tea and coffee	0.90	0.79	0.90	0.72	0.68	0.75	0.65	0.68
Salty snacks	0.18	0.48	0.23	0.14	0.30	0.35	0.32	0.25
Simple sugars	0.91	0.77	0.87	0.77	0.74	0.65	0.73	0.79
Honey and jams	0.64	0.53	0.69	0.43	0.48	0.60	0.37	0.50
Soft drinks	0.55	0.62	0.54	0.54	0.56	0.48	0.43	0.40
Snacks and desserts	0.51	0.54	0.35	0.31	0.37	0.34	0.12	0.12

Median of Spearman ​ correlation coefficients	0.53	0.53	0.43	0.44	0.45	0.48	0.43	0.37

^a^FFQ – Food Frequency Questionnaire, TLGS – Tehran Lipid and Glucose Study; FFQ1 and FFQ2 were completed 1 month before the first recall and 1 month after the 12th recall, respectively; twelve 24-hour dietary recalls were collected on a monthly basis during 12 months.

^b^All coefficients were significant (*P* < 0.05), except for liquid oil in all correlations, solid fat in adjusted correlations, and salty snacks in crude correlations of FFQ1 and adjusted correlations.

^c^All coefficients were significant (*P* < 0.05), except for legumes in crude and adjusted correlations, salty snacks in adjusted correlation of FFQ2, and snacks and deserts in adjusted correlations.

^d^Adjusted for age and total energy intake and corrected for random within-person variation.

Intraclass correlation coefficients of the 2 FFQs, adjusted for age and energy intake, are shown in Table 5. Crude correlations varied from 0.41 (salty snacks) to 0.94 (tea and coffee) in men and 0.45 (potato) to 0.83 (simple sugars and honey) in women. The correlations for all food groups were greater than 0.5, with the exceptions of solid fat, liquid oil, and salty snacks in men, and potato and nuts and seeds in women. Age- and energy-adjusted intraclass correlations ranged from 0.20 (potato) to 0.91 (tea and coffee) in men, and from 0.37 (nuts and seeds) to 0.74 (simple sugars) in women. Correlations greater than 0.5 were observed for refined grains, fruits, legumes, meats, tea and coffee, simple sugars, and soft drinks in men, and for all food groups except nuts and seeds in women.

**Table 5.** Intraclass correlation coefficients for food groups between the 2 FFQs developed for the TLGS in both sexes^a^

Food group	Men (*n* = 61)^b^		Women (*n* = 71)^c^	
	
	Crude	Adjusted^d^	Crude	Adjusted^d^
Whole grains	0.60	0.45	0.64	0.53
Refined grains	0.64	0.59	0.55	0.51
Potatoes	0.64	0.52	0.45	0.56
Dairy products	0.73	0.48	0.68	0.66
Vegetables	0.76	0.46	0.74	0.50
Fruits	0.83	0.70	0.64	0.58
Legumes	0.66	0.59	0.52	0.57
Meats	0.79	0.72	0.70	0.56
Nuts and seeds	0.58	0.34	0.46	0.52
Solid fats	0.48	0.30	0.60	0.53
Liquid oil	0.46	0.52	0.59	0.62
Tea and coffee	0.94	0.91	0.73	0.72
Salty snacks	0.41	0.28	0.68	0.70
Simple sugars	0.86	0.77	0.83	0.74
Honey and jams	0.54	0.45	0.83	0.65
Soft drinks	0.68	0.61	0.74	0.65
Snacks and desserts	0.74	0.42	0.77	0.53

Median of intraclass ​ correlation coefficients	0.66	0.52	0.68	0.57

^a^FFQ – Food Frequency Questionnaire, TLGS – Tehran Lipid and Glucose Study; FFQ1 and FFQ2 were completed 1 month before the first recall and 1 month after the 12th recall, respectively; twelve 24-hour dietary recalls were collected on a monthly basis during 12 months.

^b^All coefficients were significant (*P* < 0.05), except for nuts and seeds, solid fat, and salty snacks in adjusted correlation.

^c^All coefficients were significant (*P* < 0.05).

^d^Adjusted for age and total energy intake.

The percentages of subjects with complete agreement, adjacent agreement, and complete disagreement for the 17 food groups obtained from FFQs and 24-hour DRs are shown in Table 6. The highest percentage of complete agreement was seen for tea and coffee in FFQ1 and snacks and desserts in FFQ2 in men, and for simple sugars in FFQ1 and tea and coffee in FFQ2 in women. Regarding complete disagreement, in men the highest percentage was for dairy products (22.5) in FFQ1 and potato, dairy products, and solid fats (12.8) in FFQ2; the lowest was for vegetables, tea and coffee, and honey and jams (2.5) in FFQ1 and tea and coffee and soft drinks (0) on FFQ2. In women, the highest percentage was for solid fats (29.6) in FFQ1 and salty snacks (15.9) in FFQ2; the lowest was for simple sugars (0) in FFQ1 and tea and coffee (1.4) in FFQ2.

**Table 6.** Comparison of daily intakes of food groups based on twelve 24-hour dietary recalls and FFQs developed for the TLGS by tertile classification in both sexes^a^

Food group	Men (*n* = 61)						Women (*n* = 71)					
	
	Complete agreement (%)		Adjacent agreement (%)		Complete disagreement (%)		Complete agreement (%)		Adjacent agreement (%)		Complete disagreement (%)	
					
	FFQ1	FFQ2	FFQ1	FFQ2	FFQ1	FFQ2	FFQ1	FFQ2	FFQ1	FFQ2	FFQ1	FFQ2
Whole grains	50	55	40	35	10	10	59.1	43.7	35.3	46.6	5.6	9.8
Refined grains	55	60	40	35	5	5	35.8	54.3	51.5	40	12.8	5.7
Potatoes	45	38.5	32.5	48.8	22.5	12.8	38	46.3	45.1	39	16.9	14.4
Dairy products	55	46.1	37.5	41.1	7.5	12.8	56.3	55	42.4	34.7	1.4	10.1
Vegetables	52.5	52.5	45	45	2.5	2.5	38	46.5	47.9	45.1	14.1	8.4
Fruits	50	44.4	40	50	10	5.6	45.1	38.1	45.2	49.4	9.8	12.7
Legumes	35	34.3	47.5	55.2	17.5	10.5	40.9	44.3	46.6	40	12.9	15.7
Meats	52.5	41	37.5	51.3	10	7.7	49.3	48.6	42.3	42.9	8.4	8.6
Nuts and seeds	52.5	58	45	36.8	5	5.2	42.3	38.1	39.6	48	18.4	14.1
Solid fats	57.5	51.3	32.5	35.9	10	12.8	32.4	50	38	42.9	29.6	7.2
Liquid oil	35	53.8	50	35.9	15	10.3	50.7	55.1	40.9	41.9	8.4	2.9
Tea and coffee	77.5	53.8	20	46.1	2.5	0	45.1	62.9	43.7	35.8	11.3	1.4
Salty snacks	57.5	59.4	32.5	32.4	10	8.1	45.1	46.4	37.9	37.6	16.9	15.9
Simple sugars	62.5	52.5	30	45	7.5	2.5	60.5	52.1	39.6	45.2	0	2.8
Honey and jams	57.5	50	40	40	2.5	10	40.8	59.2	35.3	35.3	23.9	5.6
Soft drinks	55	53.8	40	46.2	5	0	51.5	46.2	38.5	43.1	10	10.5
Snacks and desserts	60	60.6	30	31.6	10	7.9	54.6	34.7	40.5	53.5	10.1	11.6

^a^FFQ – Food Frequency Questionnaire, TLGS – Tehran Lipid and Glucose Study; FFQ1 and FFQ2 were completed 1 month before the first recall and 1 month after the 12th recall, respectively; twelve 24-hour dietary recalls were collected on a monthly basis during 12 months.

## DISCUSSION

The acceptable correlations observed between FFQ2 and twelve 24-hour DRs confirmed the relative validity of the FFQ, and the good correlation observed between the 2 FFQs showed the reproducibility of the FFQ for the main food groups, including grains (whole and refined), vegetables, fruits, milk and milk products, and meat.

To our knowledge, no previous study from Iran has investigated the validity of an FFQ for foods and food groups. However, the validity and reproducibility of an FFQ for nutrients was assessed in a study conducted in Golestan with a sample size of 131 participants.^18^ Studies on the validity and reproducibility of FFQs usually focus on nutrients rather than food groups; similar studies on food groups have been performed in Denmark, German, Spain, and Sweden with 121, 104, 101, and 195 subjects, respectively.^15^^–^^17^^,^^19^

We used an age- and sex-stratified sampling method to obtain a representative sample of population. While the weighted dietary record is the gold standard used to evaluate the relative validity of an FFQ, it is more demanding and may cause individuals to change their diet.^12^ Similar to other studies, we completed 12 monthly 24-hour DRs for each participant^16^^,^^17^^,^^19^ because our population was familiar with this method^26^ and because literacy, which is essential for keeping dietary records, is not an issue in 24-hour DRs. Sources of errors using the 24-hour DRs are its reliance on memory, the lack of adequate food descriptive detail, and quantification of portion sizes used.^7^ To reduce “measurement error”, we used the same trained dietary interviewers for each participant, which helped the participants better recall their dietary intake; the use of household measures further simplified the recall process.

In our study, the FFQ was found to overestimate dairy products, nuts and seeds, and liquid oil consumption and underestimate soft drink and meat consumption, as compared with 24-hour DRs. However, other FFQ validity studies have shown both over- and underestimation of the same food groups.^16^^,^^32^^,^^33^ Usually, overestimation occurs for foods that are perceived as healthy, and underestimation occurs regarding socially unacceptable foods. Comparing the mean food group intakes from the first and second FFQ revealed that for most food groups there was no significant difference between the FFQs.

Adjusting for energy and deattenuating the validity correlation caused a decrease in almost all groups, which may be due to high between-person variation in the intake of food groups in our study subjects. The results of relative validity testing in the present study showed main food group correlations that were very similar to those noted in a Sweden population^15^—a mean (range) of 0.44 (0.15–0.69) in men and 0.49 (0.21–0.68) in women—and in a Japanese cohort,^14^ which had a mean (range) of 0.39 (0.07–0.81) in the total population. The higher correlations of food groups like whole grains, refined grains, dairy products, vegetables, fruits, meats, tea and coffee, and simple sugars may be due to more frequent consumption of these food items in our population, as compared with other food groups.^34^ Tea and coffee (the most commonly consumed drinks in Iran) and simple sugars had the highest correlations in our study, while coffee, more commonly consumed in other countries, showed the highest correlation in previous studies.^9^^,^^14^^,^^17^^,^^33^ Potatoes and legumes in both sexes, and nuts and seeds in females, showed a low correlation in our population. The European Prospective Investigation into Cancer and Nutrition (EPIC) group of Spain and Germany also reported the same results for legumes,^17^^,^^19^ while the Australian adult population and the Health Professional Follow-up Study also showed a low correlation for nuts^9^^,^^13^^,^^17^; a Japanese cohort study also reported similar correlations for potatoes.^12^ In our study, most crude intraclass correlation coefficients for both sexes exceeded 0.6, a finding in line with the results of a study that explored reproducibility in the EPIC-Heidelberg cohort,^35^ with a mean (range) of 0.51 (0.41–0.73) in men and 0.51 (0.40–0.76) in women. After adjusting for age and energy intake, correlations decreased slightly for almost all food groups, while poor reproducibility (*r* < 0.3)^36^ was seen only in males for potatoes and salty snacks.

Weak correlations are mostly seen when the frequency of consumption is low and the within-person variability is high.^33^ Portion sizes estimated for food groups with low validity in our FFQ may have caused individuals to report inaccurately their exact amount of consumption. There were several reasons for the poor reproducibility of potatoes and nuts in our FFQ: estimating the exact amount of potatoes used in food dishes is difficult, since potatoes are used with other ingredients in meal preparations (eg, in mashed potatoes served with meat, soups, etc.). The infrequent consumption of nuts and seeds in Iran, which are eaten only on formal occasions and at special ceremonies, may be the reason for the lower correlation, a finding in contrast to populations with higher consumption frequency and correlations for nuts.^37^

Males had the lowest correlation for liquid oil and solid fat, perhaps because of their lack of culinary knowledge. In addition, people use different portion sizes (eg, teaspoon, tablespoon, or cup) for liquid oils and solid fats in food preparations, so estimating the actual amount of lipids is problematic. For solid fats (butter, hydrogenated margarine, etc.) that are mostly eaten at breakfast or for snacks, we used the teaspoon as a portion size in our FFQ. Using other portion sizes, like thick, medium, or thin spread, might have yielded better results. In addition, males perceive serving sizes differently than do females.^9^

Some strengths of our study are that it included twelve 24-hour DRs administered over 12 consecutive months, which reduced the daily and seasonal variations in our study population. In addition, we deattenuated and adjusted for energy, which helped avert the random errors that result from intraperson variations. Enrolling both sexes and analyzing data for each separately are other strong points. Because of the similar distributions in sociodemographic characteristics of the participants in the present study and the cohort population, the present results can be generalized to the cohort population. Our study does, however, have some limitations. The 24-hour DR, as compared with dietary records, cannot evaluate the exact validity and relative validity of an FFQ. The small sample size of the present study kept us from categorizing subjects according to their BMI and physical activity. We used the same portion sizes for both sexes; better results could have been obtained had we used different portion sizes for males and females, considering the sex differences. Finally, using photographs to distinguish the consumed portion sizes might have yielded better results.

In conclusion, the FFQ developed for the TLGS facilitates accurate ranking of individuals according to levels of their food group intake and seems to be an acceptable tool for assessing the intake of food groups, based on its reasonable relative validity and reproducibility correlations.
